# King’s procedure for Aitken B/Paley 2a proximal femoral focal deficiency with 19-year follow-up — a case report

**DOI:** 10.3109/17453674.2013.795102

**Published:** 2013-05-31

**Authors:** Robert W Simpson-White, James A Fernandes, Michael J Bell

**Affiliations:** Sheffield Children’s NHS Trust, Western Bank, Sheffield, UK

A girl was born in 1984 with congenital anomalies in all 4 limbs. These included bilateral proximal femoral focal deficiency (PFFD), an absent right fibula, abnormal right foot and ankle, short left fibula, and rudimentary forearms with only single digits below the humerus bilaterally. The etiology remains unknown. Cerebral function was normal. Her father had spina bifida.

At the age of 1 year, she underwent a Symes amputation of the right foot and was fitted with a below-knee orthosis, with which she began to walk. During the following year, radiographs suggested a pseudarthrosis between the right femoral head and the upper femoral shaft (Figure 1). Both acetabulae were developing well, with femoral heads enlocated. This was in keeping with an Aitken type-A or type-B PFFD of the right hip (Aitken 1969). The left proximal femur, although not normal, appeared in solid continuity with the femoral head.

**Figure 1.**
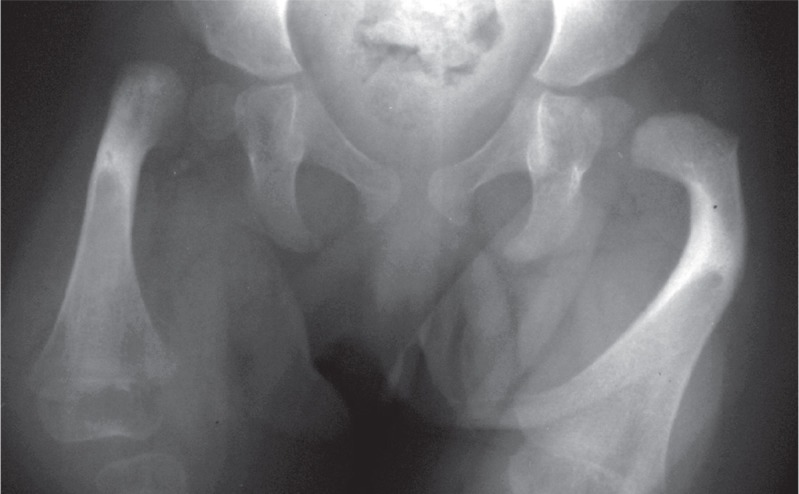
Both hips at 1 year of age.

Exploration and bone grafting of the right hip was carried out at the age of 2 years through a modified Smith-Peterson approach. At operation, there appeared to be no pseudarthrosis between the head and neck, only cartilaginous tissue at this level with a marked flexion deformity of the femur. Corticocancellous graft strips from the iliac crest were taken, laid upon the neck, and sutured in place, with the hope of encouraging ossification. In the months following this, she began to walk again without pain or dysfunction relating to the hip joint.

At the age of 5, the patient underwent a Grice procedure to arthrodese her left subtalar joint. Simultaneous examination under anesthesia of both hips was performed: there was found to be no contact between the head and upper femoral shaft on the right side. Therefore, shortly after this, the right hip was re-explored, using the previous approach. There was only fibrous tissue between the head and upper femur. It was felt that it would be impossible to perform a femoral neck reconstruction, so King’s procedure was carried out (King 1966, 1969). The upper end of the femur was sharpened and driven into a hole in the remnant of the femoral neck. The periosteal sleeve removed from the proximal femur was wrapped around this, and the construct was stabilized with a Kirschner wire (Figure 2). A full hip spica was applied.

**Figure 2.**
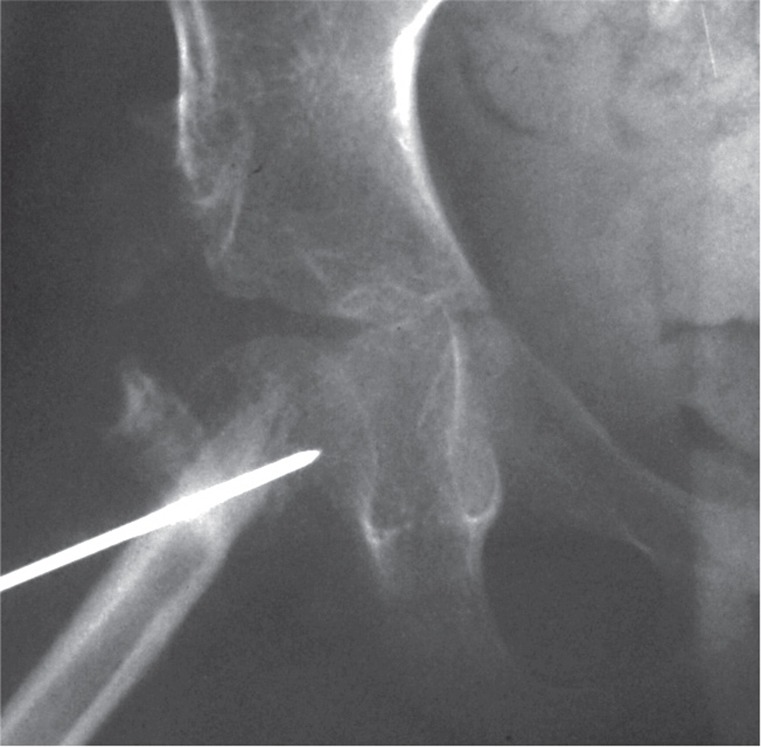
King’s procedure: immediately after surgery.

The spica was left for 3 months, after which the Kirschner wire was removed and the hip was left free. Radiographs taken at this stage showed good bone formation around the proximal femur (Figure 3). 1 year after this, the patient fell and suffered a fracture across the neck of the right femur; this was treated in a hip spica and healed without incident. She continued to walk and run with the use of an orthosis.

**Figure 3.**
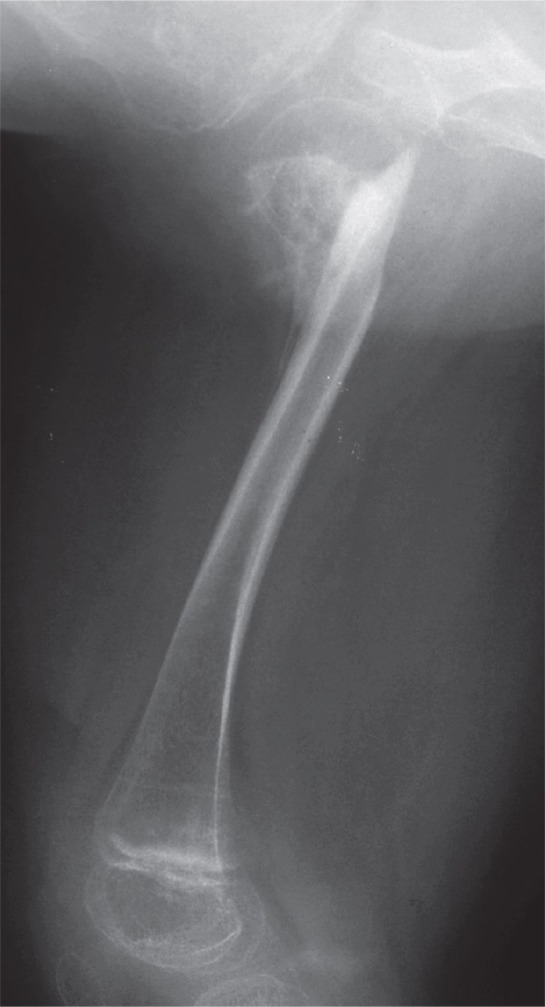
Right hip 3 months postoperatively.

Further surgical procedures included a valgus osteotomy to the left femoral neck when 8 years old for a coxa vara of 90° causing impingement, a left foot tibialis anterior transfer for a varus hindfoot at age 10, and talonavicular and calcaneocuboid arthrodeses of this foot at age 22 (effectively making a triple arthrodesis, given her previous Grice subtalar arthrodesis). She has had no surgery to her upper limbs and functions well with these.

At 24 years of age, the patient is currently able to walk 5 km with only mild-to-moderate pain in her hips. Stairs present no difficulty, and she does not use any walking aid. Running is possible for short distances, but co-ordination is difficult for this. She is gainfully employed in journalism. Current radiographs show a solid femoral neck with an enlocated hip joint (Figure 4). She has not had any surgery to the acetabulum of this hip: the radiograph shows evidence of heterotopic ossification at the superolateral margin of the hip joint.

**Figure 4.**
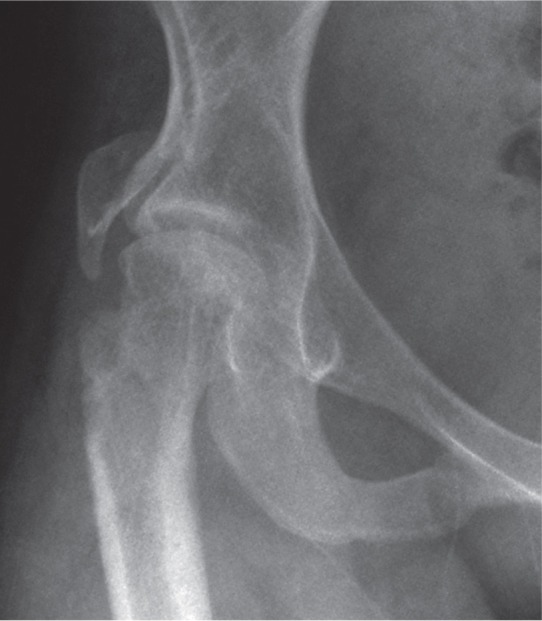
Hip 19 years postoperatively.

## Discussion

The treatment for PFFD in individual cases depends on the pattern of involvement: either by a biological reconstruction and limb lengthening strategy or ablative reconstruction with prosthetic fitment. In the latter group (those patients with severe femoral shortening and deficient hip joint), one surgical strategy described includes amputation at the level of the ankle (such as a Symes amputation), coupled with fitting of an above-knee prosthesis. The patient still lacks pelvi-femoral stability, leading to a Trendelenburg lurch (Goddard et al. 1995), and often the knee requires arthrodesis to achieve a more vertical limb. Alternatively, the limb can be rotated through a proximal tibial osteotomy (Van Ness 1950) in order for the ankle to function as a knee joint, and a below-knee prosthesis fitted to the residual foot. Potential problems include the need for multiple staged corrective surgeries (Kritter 1977), the possibility of gradual derotation with growth, and once again, lack of pelvi-femoral stability. A third possibility is to resect the residual proximal femur and thigh musculature, to rotate the limb through this defect, and to arthrodese the remaining distal femur to the pelvis (Brown 2001). Brown described this procedure to be technically demanding, although surgery can usually be performed at one sitting. Derotation is reported to be less likely to happen, pelvi-femoral stability is achieved, and patients can be fitted with a below-knee prosthesis.

Our patient did not have severe femoral shortening; therefore, treatment was aimed at biological reconstruction. The challenge was to achieve union and ossification of the femoral neck after a previous failed attempt by iliac crest strut bone grafting.

Panting and Williams (1978) reviewed a series of 23 cases. They treated pseudarthrosis of the proximal femur by exploration of the hip in 3 cases, by rodding with or without grafting of the pseudarthrosis in 2 cases, and by intramedullary nailing of the pseudarthrosis and the tibia in 1 case. The outcomes of these cases were not described in detail. Gillespie and Torode (1983) described a series of 43 patients with true PFFD. 11 of these had a defect between the head and proximal femur and underwent valgus osteotomy with or without repair of the pseudarthrosis. Many of these continued to have marked abductor weakness and Trendelenburg gait. Lloyd-Roberts and Stone (1963) described the technique of fibular grafting combined with valgus osteotomy in a case of pseudarthrosis of the proximal femur. Union was achieved, but the procedure was complicated by heterotopic ossification between the femur and the ilium; this recurred despite excision. A tibial graft was used in a second case described by the same authors, but without this complication.

Paley (1998) described the Superhip (Systematic Utilitarian Procedure for Extremity Reconstruction) procedure. His method involves adequate soft tissue release around the proximal femur coupled with simultaneous correction of the varus deformity and treatment of the pseudarthrosis by osteotomy of the proximal femur and stabilization with appropriate hardware. The hip abductors are balanced by suturing to the greater trochanter.

In King’s procedure, the sharpened proximal end of the femur is driven into the cancellous femoral neck remnant. This alters the local biomechanics, converting a shear force into a vertical compressive force and thereby promoting osteosynthesis. King (1966) described this technique in a textbook in conjunction with arthrodesis of the knee, stabilized with an intramedullary nail that exited the femur proximally (by virtue of the bow of that particular femur) and that was anchored in the pelvis, thus avoiding penetration of the femoral neck and hip joint. We were fortunate that a single Kirschner wire in combination with a hip spica provided sufficient stability until union was achieved. Our patient was not a candidate for limb lengthening strategy, so she did not require any extensive surgery apart from achieving union of the pseudarthosis to improve hip mechanics and weight bearing with the prosthesis. Fixsen and Lloyd-Roberts (1974) described their use of King’s procedure to treat 4 patients. In 2 of these, where previous grafting had failed, they used a Kirschner wire for stabilization. They did not, however, mention any long-term results.

In contrast to the Superhip procedure, King’s procedure involves less extensive surgery and it has given a satisfactory clinical outcome in this individual case.

Our case report demonstrates the appropriate use of King’s procedure to achieve proximal femoral union for pelvi-femoral stability, converting an Aitken type-B (or Paley type-2a) to Aitken type-A (or Paley type-1) PFFD. In our patient, the procedure was used as an ablative reconstructive surgery, but it could also be performed before limb lengthening in PFFD. We believe it to be the longest follow-up period for King’s procedure in the literature. It has proven to be a useful and less invasive treatment for this patient, achieving solid union sufficient for mobilization at 3 months postoperatively, and a useful hip with acceptable mobility even after 19 years.
